# Radiomics-based machine learning methods for isocitrate dehydrogenase genotype prediction of diffuse gliomas

**DOI:** 10.1007/s00432-018-2787-1

**Published:** 2019-02-04

**Authors:** Shuang Wu, Jin Meng, Qi Yu, Ping Li, Shen Fu

**Affiliations:** 1 Department of Radiation Oncology, Fudan University Shanghai Cancer Center, 270 Dong’An Road, Xuhui District, Shanghai, 200032 China; 2 Department of Oncology, Shanghai Medical College, Fudan University, Shanghai, 200032 China; 3 Department of Radiation Oncology, Shanghai Proton and Heavy Ion Center, Shanghai, 201321 China; 4 Key Laboratory of Nuclear Physics and Ion-beam Application (MOE), Fudan University, Shanghai, 200433 China; 5 Department of Radiation Oncology, Shanghai Concord Cancer Hospital, Shanghai, 200020 China

**Keywords:** Radiomics, Isocitrate dehydrogenase, Diffuse glioma, Magnetic resonance imaging, Machine learning

## Abstract

**Purpose:**

Reliable and accurate predictive models are necessary to drive the success of radiomics. Our aim was to identify the optimal radiomics-based machine learning method for isocitrate dehydrogenase (IDH) genotype prediction in diffuse gliomas.

**Methods:**

Eight classical machine learning methods were evaluated in terms of their stability and performance for pre-operative IDH genotype prediction. A total of 126 patients were enrolled for analysis. Overall, 704 radiomic features extracted from the pre-operative MRI images were analyzed. The patients were randomly assigned to either the training set or the validation set at a ratio of 2:1. Feature selection and classification model training were done using the training set, whereas the predictive performance and stability of the model were independently assessed using the validation set.

**Results:**

Random Forest (RF) showed high predictive performance (accuracy 0.885 ± 0.041, AUC 0.931 ± 0.036), whereas neural network (NN) (accuracy 0.829 ± 0.064, AUC 0.878 ± 0.052) and flexible discriminant analysis (FDA) (accuracy 0.851 ± 0.049, AUC 0.875 ± 0.057) displayed low predictive performance. With regard to stability, RF also showed high robustness against data perturbation (relative standard deviations, RSD 3.87%).

**Conclusions:**

RF is a promising machine learning method in predicting IDH genotype. Development of an accurate and reliable model can assist in the initial diagnostic evaluation and treatment planning for diffuse glioma patients.

**Electronic supplementary material:**

The online version of this article (10.1007/s00432-018-2787-1) contains supplementary material, which is available to authorized users.

## Introduction

Gliomas account for approximately 70% of malignant central nervous system (CNS) tumors (Ostrom et al. 2017). According to the 2016 World Health Organization (WHO) classification of CNS tumors, adult diffuse gliomas consist of astrocytic tumors, oligodendrogliomas, and glioblastomas (WHO grade II–IV) (Louis et al. 2016). Genomic characterization has demonstrated that identifications of mutations in isocitrate dehydrogenase (IDH) genes were associated with improved prognosis in patients with glioma (Cancer Genome Atlas Research et al. 2015; Hartmann et al. 2010; Parsons et al. 2008; Yan et al. 2009). The median overall survival of patients with IDH-mutated glioblastoma was 31 months compared to 15 months for those without the mutation (Yan et al. 2009). Patients diagnosed with IDH wild-type grade II–III glioma, which was molecularly and clinically similar to glioblastoma, had worse overall survival than those with IDH-mutated glioma of same grade (Cancer Genome Atlas Research et al. 2015). It thus seems that the identification of IDH genotype is important in the management of gliomas.

At present, the most commonly used method to assess IDH mutation status is molecular assay following biopsy or surgical resection. Although molecular assay can be informative, there are many factors that can limit its clinical use in evaluating treatment response and monitoring cancer progression (Rios Velazquez et al. 2017). These limiting factors include the lack of regular biopsies or surgical resections at the end of each treatment course, difficulty in evaluating the intra-tumor heterogeneity, inconvenient access of tumor samples, and failure to identify molecular genotype due to the poor quality of tumor tissues. In contrast to molecular assays, magnetic resonance imaging (MRI) is routinely used in the initial diagnosis and treatment response assessment of gliomas. Taking full advantage of the abundant information in these easily accessible images may provide an opportunity to overcome the limitations related to molecular assay.

MRI features have been used to predict the clinical outcomes and molecular subtypes including IDH genotype in glioma (Carrillo et al. 2012; Lee et al. 2015; Park et al. 2017). However, only a few imaging features have been used. Moreover, the identification of qualitative features is often inconsistent between observers. “Radiomics”, an emerging and promising field, hypothesizes that the advanced analysis of medical images can capture hundreds of additional features which are not currently used and may be valuable in personalized medicine (Lambin et al. 2012). Several studies have investigated the potential of these high-dimensional and minable radiomic features to noninvasively facilitate tumor detection, subtype classification, therapeutic response assessment, and prognosis prediction in multiple cancers (Aerts et al. 2014; Fehr et al. 2015; Huang et al. 2016a, b; Li et al. 2016; Nie et al. 2016; Rios Velazquez et al. 2017; Zhang et al. 2017). For gliomas, radiomic features have also been applied to predict patient survival and molecular subtypes via machine learning methods (Macyszyn et al. 2016; Rathore et al. 2016; Yu et al. 2017; Zhang et al. 2017a, b, 2018). However, the effectiveness of different radiomics-based machine learning approaches in IDH genotype prediction for patients with diffuse glioma is yet to be assessed.

Using radiomic features provided in the TCGA/TCIA repositories (Bakas et al. 2017a, b), we evaluated and compared eight classical machine learning methods in terms of their stability and performance for noninvasive and pre-operative IDH genotype prediction. A total of 126 patients with diffuse glioma were enrolled for analyses. Feature selection and classification model training were performed using the training set. The predictive performance of the model was independently tested in the validation set. Our aim was to identify the optimal and reliable IDH genotype prediction methods with the full use of MRI images.

## Materials and methods

### Data collection and patient cohort

**Fig. 1:**
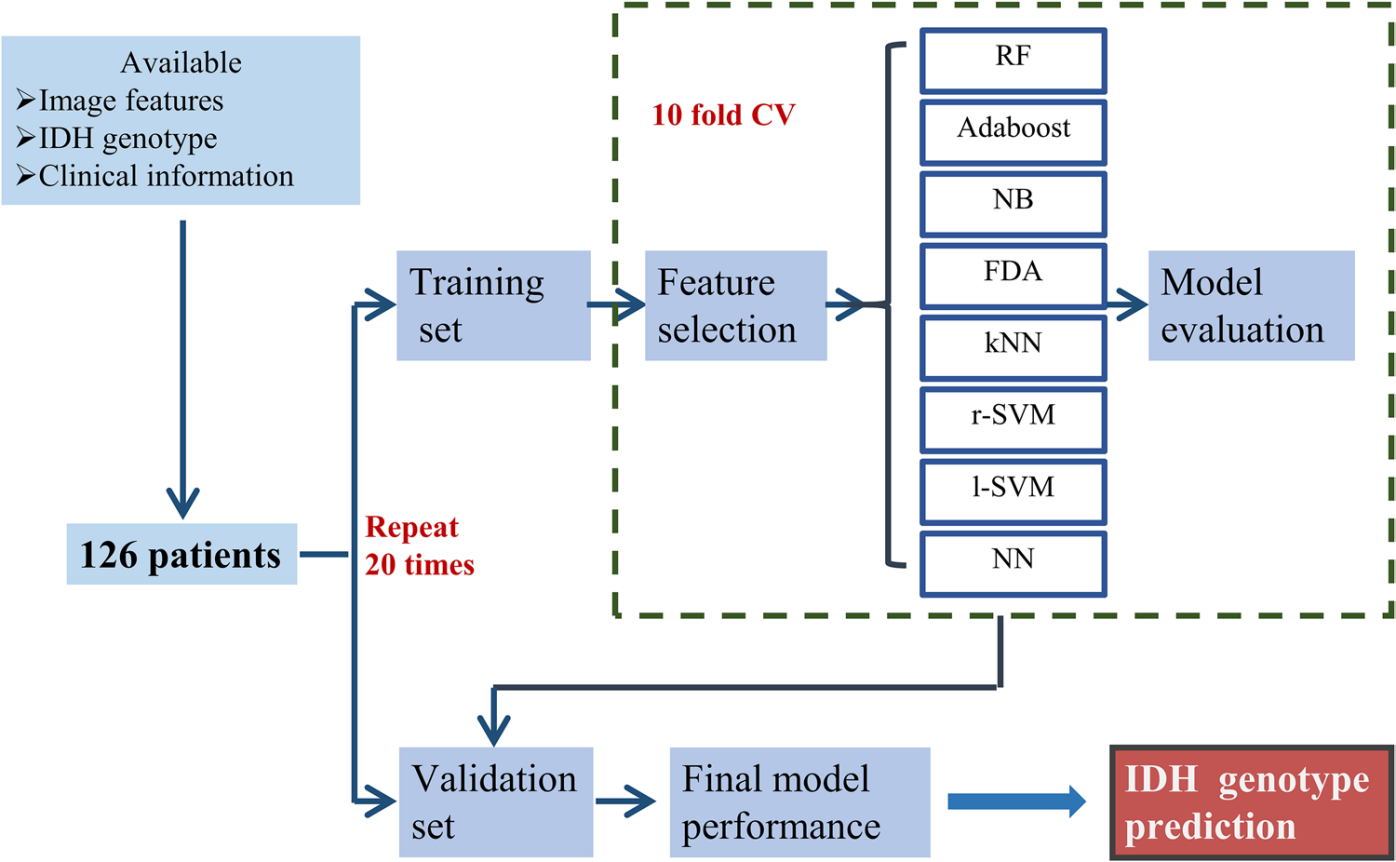
Workflow of classification method training and validation

All the patients included in our study were de-identified by the Cancer Genome Atlas; hence, no Health Insurance Portability and Accountability Act or institutional review board approval was required. Radiomic features and additional information of the TCGA-GBM and TCGA-LGG collections consisting of 102 and 65 (Bakas et al. 2017a, b) patients, respectively, were obtained from the TCIA database (Clark et al. 2013) and the related articles (Bakas et al. 2017c). IDH genotype and clinical information were acquired from a molecular profiling study of diffuse gliomas (Ceccarelli et al. 2016). We correlated the subtypes and clinical information with the imaging features using the unique TCGA identifiers. Patients who met the following criteria were included in this study: (1) available radiomic features from the enhancing part of the tumor core (ET), the non-enhancing part of the tumor core (NET), and the peritumoral edema region (ED) of pre-operative MRI images, (2) known IDH genotype, and (3) age and gender information available at diagnosis (Fig. 1). Our final cohort consisted of 126 patients with grade II–III (*n* = 43) and grade IV (*n* = 83) glioma (Table 1).

**Table 1:** Characteristics of 126 patients included in this study

	Total
Patients	*n* = 126
Histology	
Grade II–III	43 (34.1%)
Grade IV	83 (65.9%)
IDH mutation	
WT	87 (69.0%)
Mutant	39 (31.0%)
Age at diagnosis (years)	
Mean ± SD	53.5 ± 15.0
Range	18–84
Gender	
Male	67 (53.2%)
Female	59 (46.8%)

### Radiomic features

The details of image processing are described in a previous study (Bakas et al. 2017c). The pre-operative MRI modalities, which included pre-contrast T1-weighted (T1), post-contrast T1-weighted (T1-Gd), T2-weighted (T2), and T2 fluid-attenuated inversion recovery (T2-FLAIR), were used for image analysis. Three sub-regions (ET, NET, ED) were segmented by an automated method and manually revised by an experienced neuroradiologist. The intensity features, volumetric features, histogram-based features, textural parameters, spatial features, and glioma diffusion properties based on glioma growth models were calculated. The textural parameters were derived from the gray-level co-occurrence matrix (GLCM), gray-level run-length matrix (GLRLM), gray-level size zone matrix (GLSZM), and neighborhood gray-tone difference matrix (NGTDM). Finally, 704 radiomic features were extracted.

### Feature selection and classification methods

Eight classical machine learning methods were investigated and compared: Random Forest (RF), adaptive boosting (Adaboost), Naive Bayes (NB), flexible discriminant analysis (FDA), k-nearest neighbors (kNN), support vector machines with radial basis function kernel (r-SVM), support vector machines with linear kernel (l-SVM), and neural network (NN). Short descriptions of the essential features of each method are presented in Table 2.

**Table 2:** The acronyms related to the used classification methods

Acronym	Classification method name	Short descriptions
RF	Random forest	An ensemble learning method which grows substantial decision trees at training time, then combines the individual decision of each tree to obtain the optimal classification result (Liaw and Wiener 2002)
Adaboost	Adaptive boosting	A machine learning method which combines a multitude of relatively “weak classifiers” to generate a “strong classifier” (Chen and Pan 2018)
NB	Naive Bayes	A probabilistic classifier which is based on the Bayes’ theorem (Rish 2001)
FDA	Flexible discriminant analysis	A flexible extension of linear discriminant analysis, in which a classification problem is reduced to a regression problem (Hastie et al. 1994)
kNN	k-Nearest neighbors	A non-parametric learning algorithm assigning the new unknown example to the class which the majority of its “k” nearest neighbors belong to (Bishop 2006; Duda et al. 2000)
r-SVM	Support vector machines with radial basis function kernel	A classification algorithm of support vector machine using the radial basis function kernel (Cristianini and Shawe-Taylor 2000)
l-SVM	Support vector machines with linear kernel	A classification algorithm of support vector machine using the linear kernel (Cristianini and Shawe-Taylor 2000)
NN	Neural network	A computing system which is inspired by how the nervous systems in a brain works and contains a considerable amount of interconnected processing elements working in unison for solving specific problems (Bishop 2006)

Patients were randomly assigned to either the training set or the validation set at a recommended ratio of 2:1 (Dobbin and Simon 2011). With over 700 radiomic features used for analyses, it was necessary to perform feature selection before classification model building, and minimum redundancy maximum relevance (MRMR) was applied to select the relevant and non-redundant features (De Jay et al. 2013; Ding and Peng 2005). Subsequently, the selected features were input as predictors for classification models constructing. Each model was trained using repeated (5 repeat iterations) tenfold cross-validation of the training set. The predictive performance was independently estimated in the validation set by quantifying the accuracy and area under the receiver operator characteristic (ROC) curve (AUC) (Robin et al. 2011). All the machine learning algorithms were conducted using the R caret package (Kuhn 2008). The parameters set for model training are listed in Supplementary Table 1.

We repeated the procedure of training and validation 20 times to achieve a robust estimate of the model performance. Different patients were assigned to the training and the validation set each time. The stability of each machine learning method was quantified through relative standard deviations (RSD) (Parmar et al. 2015). RSD% is defined as:

$${\text{RSD\% ~}}={\text{~}}{\raise0.7ex\hbox{${\sigma \_{\text{AUC}}}$} \!\mathord{\left/ {\vphantom {{\sigma \_{\text{AUC}}} {\sigma \_{\text{AUC~}}}}}\right.\kern-0pt}\!\lower0.7ex\hbox{${\upmu \_{\text{AUC~}}}$}}\, \times \;100{\text{\% ,}}$$

where σ_AUC is the standard deviation of the 20 AUC values and µ_AUC is the mean of the 20 AUC values. A lower RSD value corresponds to higher stability of the machine learning method.

### Classification models based on features of different subcategories

To further explore the predictive values of different features in IDH genotype discrimination, the radiomic features were classified into six subcategories. The definitions of these features’ subcategories are shown in Table 3. Feature selection was performed for each subcategory except spatial and TGM, as these two had few features. The predictive performance of the final model was independently estimated using the accuracy and AUC in the validation set. To achieve an average estimate of the model performance, we repeated the procedure 20 times.

**Table 3:** Table defining the feature subcategory

Feature subcategory	Feature subcategory definition	Feature number
Volume	Features of volumetric type	21
Spatial	Features of spatial type	11
TGM	Tumor growth model parameter	6
NET-hit	Histogram-based, intensity and textural features of the non-enhancing part of the tumor core	222
ET-hit	Histogram-based, intensity and textural features of the enhancing part of the tumor core	222
ED-hit	Histogram-based, intensity and textural features of the peritumoral edema	222
ALL	All features	704

All the analyses were completed using the R software (version 3.3.1).

## Results

### Predictive performance of the classification methods

In the current study, 126 patients (Table 1) were enrolled to investigate the effectiveness of eight machine learning methods (Table 2) for IDH genotype prediction. The workflow of classification model training and validation is displayed in Fig. 1. The accuracy and AUC were calculated to quantify the predictive performance of the classification methods. A total of 704 radiomic features were filtered by the feature selection method, MRMR. Then, the top 5, 10, 15, 20, 25, 30, 35 and 40 selected features were used to train the classifiers separately. The mean AUC and accuracy of all the classification methods trained with the selected features are reported in Supplementary Table 2 and Supplementary Table 3. In terms of accuracy, RF with top 20 selected features showed the highest predictive performance (accuracy 0.895 ± 0.043, range 0.825–0.975), whereas NN with top 40 selected features displayed the lowest predictive performance (accuracy 0.829 ± 0.064, range 0.659–0.927) (Supplementary Table 2). As far as the AUC values were concerned, RF with top 15 selected features had the highest predictive performance (AUC 0.931 ± 0.036, range 0.866–1), while FDA with top 25 selected features showed the lowest predictive performance (AUC 0.875 ± 0.057, range 0.761–0.983) (Supplementary Table 3).

### Stability of the classification methods

**Fig. 2:**
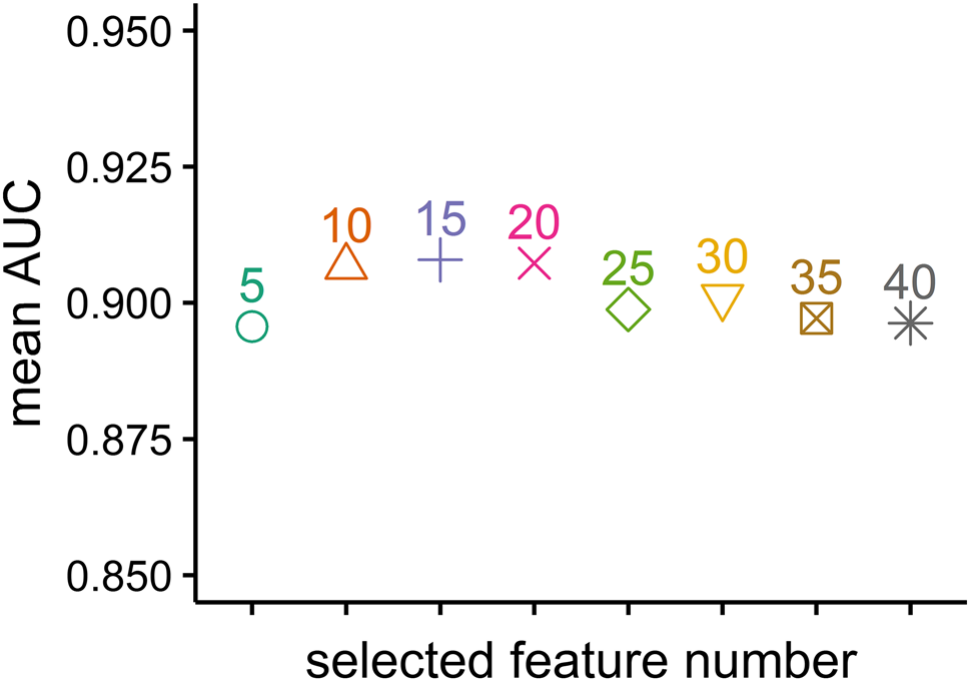
Mean AUC values over all classifiers

**Fig. 3:**
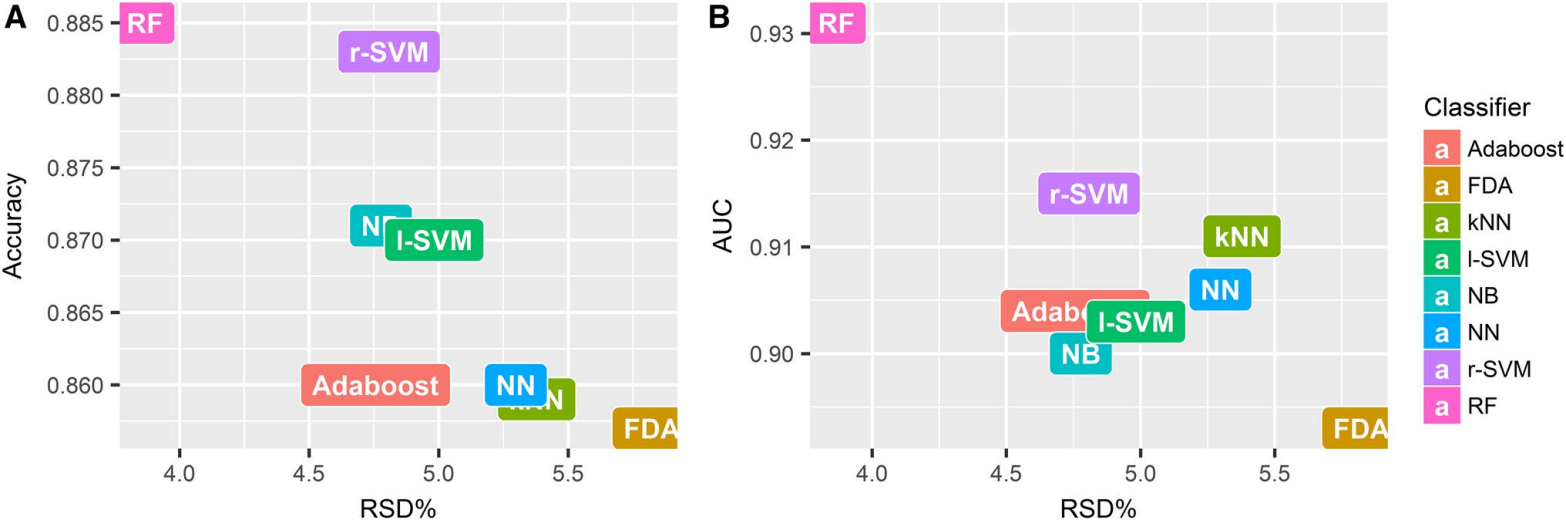
Plots between model stability and predictive performance (Accuracy, AUC) of classifiers with top 15 selected features. **a** Accuracy vs RSD. **b** AUC vs RSD. *RSD* relative standard deviation; *AUC* area under receiver operator characteristics curve

As the mean AUC value over all classifiers was highest when top 15 selected features were used (Fig. 2), the stability of classification methods with top 15 selected features was analyzed. The most stable classification model was RF (RSD = 3.87%), followed by Adaboost (RSD = 4.76%), NB (RSD = 4.78%) and r-SVM (RSD = 4.81%). FDA (RSD = 5.82%) and kNN (RSD = 5.34%) had the lowest stability among all the classification methods. Figure 3 shows an evaluation of model stability and predictive performance. We observed that RF (RSD = 3.87%, AUC 0.931 ± 0.036, accuracy 0.885 ± 0.041) out-performed other machine learning methods.

### Classification models based on features of different subcategories

**Fig. 4:**
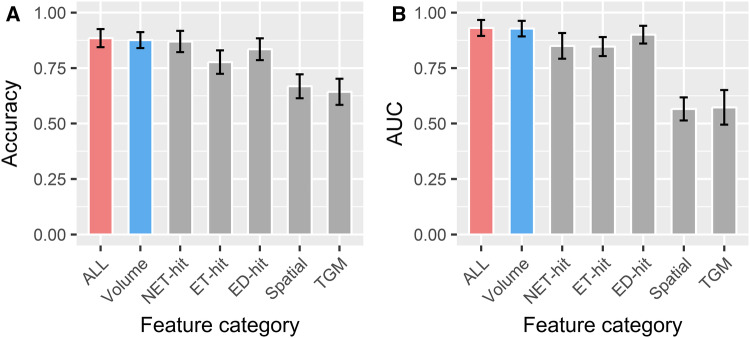
Predictive performance of RF based on different feature subcategory. The top 15 selected features were used to train RF. **a** Accuracy; **b** AUC

To investigate the value of the different features in IDH genotype predicting, the features were divided into six subcategories (Table 3). As RF had the highest stability and predictive performance among the eight classifiers, we used it to estimate the predictive role of different subcategories. As illustrated in Fig. 4, volume, NET-hit, ET-hit, and ED-hit out-performed spatial and TGM. The RF model based on volume features (AUC 0.928 ± 0.035, range 0.838–1; accuracy 0.876 ± 0.036, range 0.805–0.927) showed the highest predictive performance, whereas the RF model based on spatial features (AUC 0.566 ± 0.052, range 0.468–0.664, accuracy 0.668 ± 0.054, range 0.585–0.756) or TGM features (AUC 0.573 ± 0.078, range 0.424–0.743, accuracy 0.643 ± 0.059, range 0.537–0.756) had the lowest predictive performance.

## Discussion

Precision oncology refers to customizing cancer care for individual patients. Such individual customization can maximize the benefits of prevention and treatment interventions while minimizing adverse effects. The success of precision oncology relies on accurately categorizing patients on the basis of their prognostic characteristics and responses to a specific treatment. As quantitative features extracted from medical images can be used to enhance the understanding of tumor characteristics, some studies have explored the value of radiomic features in precision oncology (Aerts et al. 2014; Nie et al. 2016; Rios Velazquez et al. 2017). Rios Velazquez et al. illustrated that radiomics-based machine learning model can be used to predict EGFR mutation status, which is an important biomarker for the treatment of lung cancer (Rios Velazquez et al. 2017). Nie et al. showed the potential of radiomics-based machine learning model to predict pathologic responses after pre-operative chemoradiation therapy for locally advanced rectal cancer (Nie et al. 2016). Results from these studies suggested that highly accurate and reliable classification models can promote the success of radiomics in precision oncology. Furthermore, identifying the optimal machine learning methods is recommended for different clinical tasks (Lambin et al. 2017).

Parmar et al. compared 12 machine learning methods in terms of their prognostic performance and stability for overall survival (OS) prediction in patients with lung cancer. They identified Random Forest (AUC: 0.66 ± 0.03) as the method with the highest prognostic performance and high stability (Parmar et al. 2015). Zhang et al. evaluated nine classification methods in terms of their predictive performance for the prediction of local failure and distant failure in advanced nasopharyngeal carcinoma. Random Forest (AUC 0.85 ± 0.01) and adaptive boosting (AUC 0.82 ± 0.01) were found to have the highest prognostic performance (Zhang et al. 2017). In another study, Parmar et al. investigated 11 machine learning methods in terms of their performance for predicting OS in patients with head and neck cancer. Bayesian (AUC 0.67, RSD: 11.28), Random Forest (AUC 0.61, RSD 7.36), and Nearest Neighbor (AUC 0.62, RSD 10.52) displayed high prognostic performance and stability (Parmar et al. 2015a, b). However, the optimal machine learning methods for IDH genotype predicting in patients with diffuse glioma have yet to be determined.

In the present study, we investigated and compared eight radiomics-based machine learning methods to pre-operatively predict IDH genotype for diffuse gliomas. As described in the previous study, MRI images used for feature extraction were collected from eight institutes, which may make the model broadly applicable in the clinical practice (Bakas et al. 2017c). Moreover, the features analyzed in this study were extracted from labels segmented through a semi-automatic approach (Bakas et al. 2017c), which can reduce the variation of delineation between different observers and produce more reproducible and stable features (Parmar et al. 2014). More stable features may result in a more reliable classification model.

As over 700 quantitative radiomic features were analyzed in the current study, feature selection was performed. Feature selection is an exceedingly helpful strategy in data mining. It can help simplify the model, avoid the curse of dimensionality, and reduce over-fitting. MRMR was applied for feature selection in our analyses (Ding and Peng 2005). Finally, the top 5, 10, 15, 20, 25, 30, 35 and 40 selected features were used to train the classifiers separately. The accuracy, AUC, and RSD were quantified to evaluate the predictive performance and stability of eight classical machine learning methods. The average performance over all classifiers was highest when the top 15 selected features were used, and RF (AUC 0.931 ± 0.036, accuracy 0.885 ± 0.041, RSD 3.867) had the highest predictive performance and stability. As one of the most frequently used machine learning algorithms in clinical classification problems (Parmar et al. 2015; Rios Velazquez et al. 2017; Zhang et al. 2017a, b), RF reduces over-fitting by bootstrap sampling and randomly selecting features at each split in the process of training (Liaw and Wiener 2002). The results from our analyses suggest that RF should be preferred with regard to predicting IDH genotype for patients with gliomas.

In the current study, we also evaluated and compared the predictive value of different feature subcategories. It has been shown that volume features were associated with molecular subtypes in glioma. Park et al. have shown that the IDH-mutant group had a smaller proportion of enhancing tumors in grade II and III gliomas (Park et al. 2017). Carrillo et al. demonstrated that the presence of non-contrast enhancing tumor was related to IDH mutation in grade IV gliomas (Carrillo et al. 2012). Textural features, which can quantify the intra-tumor heterogeneity by evaluating the gray-level intensity and position of the pixels within an image (Castellano et al. 2004; O’Connor et al. 2015), have also been applied to predict MGMT methylation status (Xi et al. 2017), EGFR expression (Li et al. 2018), and immune cell infiltration status (Narang et al. 2017) for gliomas. In our analysis, the feature subcategories volume, NET-hit, ET-hit, and ED-hit had high predictive performance (Fig. 4). Some of the patients with diffuse glioma did not demonstrate enhancement or edema on MRI scans. The models based on volume features or NET-hit features provide an opportunity to noninvasively and pre-operatively predict IDH genotype for these patients.

This study has some limitations. First, larger sample sizes and external validation are required to assess the generalizability of our model. In the current study, we repeated the training procedure 20 times; each time different patients were assigned to the training and the validation set. Furthermore, the model predictive performance was repeatedly and independently evaluated in the validation set. These approaches enabled a proper estimation of our model generalizability. Second, recent studies have illustrated that diffusion-weighted imaging and magnetic resonance spectroscopy (MRS) have the potential to noninvasively identify IDH genotype for gliomas (Branzoli et al. 2017; Choi et al. 2016; Lee et al. 2015; Zhang et al. 2017a, b). The addition of imaging features from these modalities may improve the classification performance of our model. Third, the underlying biological mechanisms of how these features are correlated with IDH genotype in diffuse gliomas are presently unclear. Further research is needed to explore these mechanisms.

In summary, the role of MRI radiomic features in IDH genotype predicting and eight radiomics-based machine learning methods was compared and investigated in the present study. RF with top 15 selected features showed the highest predictive performance and stability (accuracy 0.885 ± 0.041, AUC 0.931 ± 0.036, RSD 3.87%). These radiomics-based models maximized the value of the information contained in the medical images. Identification of an optimal radiomics-based machine learning method to noninvasively and pre-operatively predict IDH genotype can be valuable in the initial diagnostic evaluation and treatment planning for patients with diffuse glioma.

## Electronic supplementary material

Below is the link to the electronic supplementary material.

Supplementary material 1 (DOCX 24 KB)
