# Supplementary material for: Radiomics-based machine learning methods for isocitrate dehydrogenase genotype prediction of diffuse gliomas
Source: J Cancer Res Clin Oncol. 2019 Feb 4;145(3):543–50. doi: 10.1007/s00432-018-2787-1 (PMC6394679; doi:10.1007/s00432-018-2787-1)
Supplement: Supplementary file 1 — Supplementary material 1 (DOCX 24 KB) [file 432_2018_2787_MOESM1_ESM.docx]

**Supplementary Table 1.** Tuning parameter for each classifier. seq(n1, n2, by=n3), n1-3 stand for numbers, from n1 to n2 with an increase of n3

| Classifier | Method in caret | Parameter | Tuning parameter value |
| --- | --- | --- | --- |
| RF | "rf" | ntree | 1000 |
|  |  | mtry | seq(2, 30, by=2) |
| Adaboost | "adaboost" | nIter | seq(10, 80, by=10) |
|  |  | method | "Adaboost.M1" |
| NB | "naive_bayes" | usekernel | by default |
| FDA | "fda" | nprune | seq(2, 20, by = 2) |
| kNN | "knn" | k | seq(5, 35, by = 3) |
| r-SVM | "svmRadial" | C | 0.25, 0.5, 1, 2, 4, 8, 16, 32 |
|  |  | sigma | seq(0.01, 0.15, by = 0.02) |
| l-SVM | "svmLinear" | C | 0.25, 0.5, 1, 2, 4, 8, 16, 32 |
| NN | "nnet" | size | seq(1, 9, by = 2) |
|  |  | decay | 0, 10^-1^, 10^-2^, 10^-3^, 10^-4^ |

**Supplementary Table 2.** Accuracy of different classifiers using different selected feature numbers

| Classifers | 5 | 10 | 15 | 20 | 25 | 30 | 35 | 40 |
| --- | --- | --- | --- | --- | --- | --- | --- | --- |
| RF | 0.869 ± 0.04  [0.8, 0.95] | 0.89 ± 0.044  [0.8, 0.975] | 0.885 ± 0.041  [0.805, 0.951] | 0.895 ± 0.043  [0.825, 0.975] | 0.894 ± 0.041  [0.805, 0.976] | 0.894 ± 0.038  [0.825, 0.975] | 0.891 ± 0.041  [0.805, 0.976] | 0.894 ± 0.038  [0.829, 0.976] |
| Adaboost | 0.861 ± 0.043  [0.78, 0.951] | 0.868 ± 0.036  [0.805, 0.927] | 0.86 ± 0.054  [0.707, 0.927] | 0.859 ± 0.044  [0.756, 0.927] | 0.865 ± 0.048  [0.78, 0.951] | 0.861 ± 0.048  [0.756, 0.951] | 0.863 ± 0.04  [0.805, 0.951] | 0.868 ± 0.041  [0.78, 0.951] |
| NB | 0.87 ± 0.038  [0.805, 0.951] | 0.874 ± 0.037  [0.805, 0.951] | 0.871 ± 0.044  [0.805, 0.951] | 0.874 ± 0.042  [0.805, 0.951] | 0.878 ± 0.034  [0.829, 0.951] | 0.872 ± 0.033  [0.829, 0.951] | 0.882 ± 0.043  0.805 ± 0.951 | 0.879 ± 0.04  [0.829, 0.951] |
| FDA | 0.878 ± 0.049  [0.78, 0.976] | 0.852 ± 0.036  [0.78, 0.902] | 0.857 ± 0.043  [0.805, 0.951] | 0.861 ± 0.055  [0.732, 0.951] | 0.851 ± 0.049  [0.78, 0.951] | 0.86 ± 0.042  [0.78, 0.927] | 0.867 ± 0.052  [0.78, 0.976] | 0.861 ± 0.051  [0.78, 0.976] |
| kNN | 0.86 ± 0.037  [0.805, 0.951] | 0.865 ± 0.035  [0.805, 0.951] | 0.859 ± 0.036  [0.829, 0.951] | 0.862 ± 0.043  [0.829, 0.976] | 0.849 ± 0.033  [0.78, 0.902] | 0.845 ± 0.036  [0.78, 0.902] | 0.852 ± 0.038  [0.78, 0.927] | 0.84 ± 0.044  [0.756, 0.927] |
| r-SVM | 0.876 ± 0.044  [0.805, 0.951] | 0.874 ± 0.036  [0.805, 0.951] | 0.883 ± 0.046  [0.78, 0.951] | 0.884 ± 0.039  [0.78, 0.951] | 0.885 ± 0.039  [0.78, 0.951] | 0.882 ± 0.046  [0.78, 0.951] | 0.883 ± 0.049  [0.756, 0.951] | 0.885 ± 0.047  [0.756, 0.951] |
| l-SVM | 0.882 ± 0.039  [0.829, 0.951] | 0.876 ± 0.045  [0.805, 0.951] | 0.87 ± 0.054  [0.707, 0.951] | 0.861 ± 0.048  [0.732, 0.951] | 0.854 ± 0.034  [0.78 , 0.902] | 0.851 ± 0.047  [0.707, 0.927] | 0.841 ± 0.04  [0.756, 0.902] | 0.839 ± 0.043  [0.732, 0.902] |
| NN | 0.866 ± 0.055  [0.756, 0.951] | 0.845 ± 0.047  [0.78, 0.927] | 0.86 ± 0.057  [0.707, 0.951] | 0.859 ± 0.052  [0.732, 0.951] | 0.85 ± 0.051  [0.707, 0.927] | 0.834 ± 0.059  [0.683, 0.902] | 0.83 ± 0.065  [0.659, 0.927] | 0.829 ± 0.064  [0.659, 0.927] |

All these parameters were quantified using the validation set. The average performance of the classification methods is presented with standard deviation in the parenthesis, min and max values in the brackets

**Supplementary Table 3.** AUC value of different classifiers using different selected feature numbers

| Classifers | 5 | 10 | 15 | 20 | 25 | 30 | 35 | 40 |
| --- | --- | --- | --- | --- | --- | --- | --- | --- |
| RF | 0.916 ± 0.039  [0.826, 0.985] | 0.929 ± 0.036  [0.844, 0.997] | 0.931 ± 0.036  [0.866, 1] | 0.931 ± 0.037  [0.865, 0.997] | 0.922 ± 0.04  [0.856, 0.997] | 0.931 ± 0.041  [0.853, 0.994] | 0.923 ± 0.037  [0.874, 1] | 0.924 ± 0.036  [0.859, 1] |
| Adaboost | 0.883 ± 0.048  [0.802, 0.981] | 0.896 ± 0.046  [0.828, 0.994] | 0.904 ± 0.043  [0.816, 0.98] | 0.9 ± 0.037  [0.826, 0.986] | 0.904 ± 0.043  [0.813, 0.983] | 0.907 ± 0.05  [0.793, 0.983] | 0.903 ± 0.056  [0.754, 0.989] | 0.9 ± 0.055  [0.764, 1] |
| NB | 0.89 ± 0.048  [0.802, 0.997] | 0.908 ± 0.05  [0.802, 0.997] | 0.9 ± 0.043  [0.836, 1] | 0.9 ± 0.042  [0.838, 0.997] | 0.897 ± 0.046  [0.816, 1] | 0.898 ± 0.041  [0.839, 1] | 0.901 ± 0.046  [0.822, 1] | 0.899 ± 0.042  [0.822, 1] |
| FDA | 0.891 ± 0.05  [0.822, 0.994] | 0.892 ± 0.062  [0.747, 0.968] | 0.893 ± 0.052  **[**0.813, 0.986] | 0.897 ± 0.062  [0.764, 1] | 0.875 ± 0.057  [0.761, 0.983] | 0.889 ± 0.047  [0.813, 0.98] | 0.885 ± 0.047  [0.813, 0.98] | 0.882 ± 0.059  [0.763, 0.997] |
| kNN | 0.882 ± 0.053  [0.786, 0.97] | 0.902 ± 0.052  [0.807, 0.996] | 0.911 ± 0.049  [0.809, 1] | 0.902 ± 0.046  [0.789, 0.997] | 0.894 ± 0.055  [0.79, 0.999] | 0.899 ± 0.053  [0.779, 1] | 0.896 ± 0.048  [0.806, 0.997] | 0.897 ± 0.048  [0.812, 0.997] |
| r-SVM | 0.907 ± 0.046  [0.839, 0.997] | 0.913 ± 0.042  [0.83, 0.997] | 0.915 ± 0.044  [0.828, 1] | 0.912 ± 0.043  [0.828, 0.997] | 0.911 ± 0.044  [0.819, 1] | 0.909 ± 0.049  [0.805, 1] | 0.909 ± 0.046  [0.799, 1] | 0.913 ± 0.049  [0.805, 1] |
| l-SVM | 0.898 ± 0.04  [0.845, 0.991] | 0.907 ± 0.049  [0.807, 0.994] | 0.903 ± 0.045  [0.822, 0.966] | 0.909 ± 0.041  [0.83, 0.977] | 0.897 ± 0.045  [0.799, 0.963] | 0.887 ± 0.044  [0.79, 0.954] | 0.879 ± 0.043  [0.793, 0.96] | 0.877 ± 0.047  [0.77, 0.957] |
| NN | 0.898 ± 0.044  [0.807, 0.989] | 0.906 ± 0.052  [0.809, 1] | 0.906 ± 0.048  [0.825, 0.971] | 0.907 ± 0.042  [0.828, 0.98] | 0.89 ± 0.054  [0.799, 0.968] | 0.888 ± 0.045  [0.799, 0.96] | 0.881 ± 0.048  [0.799, 0.974] | 0.878 ± 0.052  [0.793, 0.991] |

AUC: area under receiver operator characteristics curve. All these parameters were quantified using the validation set. The average performance of the classification methods is presented with standard deviation in the parenthesis, min and max values in the brackets
